# Serum human epididymis protein 4, endocan, sialyl-Tn, and neopterin in endometrial cancer: a prospective case–control study

**DOI:** 10.1590/1806-9282.20251949

**Published:** 2026-06-15

**Authors:** Atahan Toyran, Yagmur Minareci, Hamdullah Sözen, Canan Kucukgergin, Abdurrahman Fatih Aydın, İlknur Bingul, Mehmet Guven Gunver, Serra Zeynep Akkoyunlu, Samet Topuz, Mehmet Yavuz Salihoglu

**Affiliations:** 1 Istanbul University, Istanbul Faculty of Medicine, Department of Gynecology and Obstetrics, Division of Gynecologic Oncology – Istanbul, Turkey.; 2 Istanbul University, Istanbul Faculty of Medicine, Department of Medical Biochemistry – Istanbul, Turkey.; 3 Istanbul University, Istanbul Faculty of Medicine, Department of Biostatistics – Istanbul, Turkey.

**Keywords:** ESM1 protein, Endometrial neoplasms, HE4 protein, human, Neopterin, Sialyl-Tn antigen

## Abstract

**OBJECTIVE:**

The aim of this study was to compare serum levels of endocan, human epididymis protein 4, sialyl-Tn antigen, and neopterin between women with endometrial cancer and asymptomatic healthy controls, and to explore their discriminatory performance in this case–control setting and clinicopathological associations.

**METHODS:**

Prospective case–control study of 133 women with endometrial cancer and 65 healthy controls. Serum biomarkers were measured before treatment by enzyme-linked immunosorbent assays. Discrimination was evaluated using the area under the receiver operating characteristic curve with optimal cut-offs.

**RESULTS:**

Endocan levels were significantly higher in endometrial cancer patients than in controls (p<0.001), with an area under the curve of 0.758. Serum human epididymis protein 4 was also elevated in patients (p<0.001), with an area under the curve of 0.702. Neopterin and sialyl-Tn did not differ significantly between groups. Endocan and human epididymis protein 4 were higher in women with lymphovascular space invasion (both p<0.05); however, these associations did not remain significant after correction for multiple comparisons.

**CONCLUSION:**

Serum endocan and human epididymis protein 4 were elevated in endometrial cancer patients compared to asymptomatic healthy controls. Neopterin and sialyl-Tn showed no discriminatory value in this setting. Our findings should be interpreted as exploratory and require validation in clinically relevant cohorts.

## INTRODUCTION

Endometrial cancer ranks as the sixth most frequently diagnosed cancer in women globally and is the second most prevalent gynecologic malignancy^1,2^. The prognosis for endometrial malignancies is generally favorable when detected at an early stage^3^. However, no reliable serum biomarkers are currently available for the early clinical identification of endometrial cancers^4^. Although the diagnosis of endometrial cancer relies on histopathological evaluation of endometrial tissue, there remains interest in circulating biomarkers as adjunct tools for preoperative risk stratification and prognostic assessment^3,4^.

Endocan (endothelial cell-specific molecule-1) is a chondroitin-dermatan sulfate proteoglycan primarily found in vascular endothelial cells, playing a key role in angiogenesis and inflammation^5^. Human epididymis protein 4 (HE4) is the most extensively studied serum marker in endometrial cancer; contemporary reviews and meta-analyses report moderate diagnostic accuracy and prognostic associations, yet clinical adoption remains limited^6,7,8^. Sialyl-Tn (STN) is a carbohydrate antigen involved in mucin production, which plays a significant role in the carcinogenesis of various malignancies^9^. Neopterin is a pteridine derivative biosynthetically produced from guanosine triphosphate in response to immune activation, particularly via interferon-gamma^10^.

Despite promising data on these emerging biomarkers, none have yet achieved widespread clinical application in endometrial cancer. Therefore, this prospective case–control study aimed to compare serum levels of endocan, HE4, STN, and neopterin between women with endometrial cancer and asymptomatic healthy controls, to describe their discriminatory performance and to investigate associations with clinical and pathological features of the disease.

## METHODS

This prospective case–control study was conducted at the Department of Gynecologic Oncology, Istanbul University, Istanbul Faculty of Medicine, a European Society of Gynaecological Oncology (ESGO)-accredited centre for endometrial cancer surgery. The study protocol was approved by the institutional ethics committee (approval number: 2019/355). Written informed consent was obtained from all participants prior to enrollment. The study complied with the Declaration of Helsinki.

The primary endpoint was the comparison of serum endocan, HE4, neopterin, and STN levels between women with endometrial cancer and asymptomatic healthy controls. Secondary endpoints were associations of these biomarkers with clinical variables and pathological features.

### Study group and sample size

A total of 133 patients with endometrial cancer and 65 asymptomatic healthy women were included in the study. Asymptomatic controls were prospectively recruited from women attending routine gynecologic visits at the same institution during the same time period, and age-based frequency matching was used to obtain a similar age distribution between groups. Sample size was estimated using G*Power (version 3.1) assuming α=0.05 and 80% power. Because clinically meaningful thresholds for these biomarkers are not established and available prior data are limited, we used a moderate standardized effect size (Cohen’s d=0.5) as a pragmatic feasibility assumption to power the primary case–control comparisons. This yielded a minimum total sample size of 128; the final cohort exceeded this minimum^11,12^. Tumor staging was performed according to the 2009 International Federation of Gynecology and Obstetrics (FIGO) classification system.

Inclusion criteria for patients were histologically confirmed endometrial carcinoma at any FIGO stage and availability of pre-treatment serum. We excluded synchronous malignancies, non-epithelial uterine tumors, recurrent disease, any prior systemic therapy before blood draw, and active infectious/autoimmune/inflammatory conditions that could influence biomarkers. Controls were prospectively recruited from routine gynecologic visits at the same institution, asymptomatic, with no history of malignancy or hysterectomy, and documented menopausal status. To minimize HE4 confounding, controls with renal impairment (estimated glomerular filtration rate<60 mL/min/1.73 m^2^ or documented chronic kidney disease stage ≥3) were excluded. Additional exclusions mirrored patients (recent infection ≤4 weeks, pregnancy, current corticosteroid/immunomodulator use, or exogenous hormone therapy within ≤3 months).

### Statistical analyses

All statistical analyses were performed using International Business Machines Statistical Package for the Social Sciences Statistics for Windows, version 30.0 (IBM Corp., Armonk, NY, USA). Continuous data are presented as mean±standard deviation or median (interquartile range [IQR]); categorical data as counts (%). Normality was assessed by the Shapiro-Wilk test. Group comparisons used t-test or Mann-Whitney U, as appropriate; biomarker analyses used non-parametric tests. Where significant, Hodges-Lehmann estimates and effect sizes (r) were reported. Diagnostic performance for endocan and HE4 was evaluated by receiver operating characteristic (ROC) analysis with area under the curve (AUC), optimal cut-off, sensitivity, and specificity. A two-tailed p<0.05 was considered statistically significant. For the exploratory subgroup comparisons presented in Table 1, p-values were adjusted for multiple comparisons using the Benjamini-Hochberg false discovery rate (FDR) across 12 tests.

**Table 1:** Association between Endocan, human epididymis protein 4, Neopterin, sialyl-Tn levels, and pathologic features of patients with endometrial cancer.

	n	Endocan pg/mL median (IQR)	p	n	HE4 pg/mL median (IQR)	p	n	Neopterin nmol/L median (IQR)	p	n^‡^	STN U/mL median (IQR)	p
Grade			0.946			0.884			0.948			0.724
Low grade (Grade 1)	102	167.1 (110.1– 267.9)		102	454.1 (262.8– 747.5)		102	3.90 (3.50– 4.72)		100	9.30 (8.34– 10.77)	
High grade (Grade 2–3)	31	147.3 (107.3– 297.6)		31	402.6 (232.3– 1,097.0)		31	3.89 (3.48– 6.16)		30	9.27 (7.96– 10.77)	
LVSI^§^			**0.020**			**0.021**			0.540			0.320
Positive	32	207.2 (123.8– 313.6)		32	454.1 (336.5– 1,290.5)		32	4.04 (1.88– 6.20)		30	9.30 (6.74– 11.86)	
Negative	101	147.5 (107.1– 262.1)		101	405.4 (247.7– 729.8)		101	3.89 (2.64– 5.14)		100	9.30 (6.68– 11.92)	
FIGO stage			0.975			0.851			0.554			0.769
Early stage (Stage I–II)	112	159.2 (109.1– 284.1)		112	454.1 (262.1– 778.6)		112	3.94 (3.52– 4.82)		110	9.30 (8.22– 10.76)	
Advanced stage (Stage III–IV)	21	160.2 (121.9– 228.3)		21	402.1 (233.7– 1,271.8)		21	3.68 (3.40– 5.82)		20	9.46 (8.37– 10.78)	

HE4: human epididymis protein 4; STN: sialyl-Tn; LVSI: lymphovascular space invasion; IQR: interquartile range; FIGO: International Federation of Gynecology and Obstetrics.

^§^For LVSI comparisons, Hodges-Lehmann median difference (95%CI) and effect size r were as follows: Endocan +54.6 (7.4–105.7), r=0.20; HE4+160.9 (25.3–334.9), r=0.20. After Benjamini-Hochberg FDR correction across 12 comparisons, q=0.126 for Endocan-LVSI and q=0.126 for HE4-LVSI; no comparisons remained significant at q <0.05.

^‡^Differences in n values across markers reflect missing data due to assay limitations. Bold values indicate statistical significance.

### Sampling and evaluation of biomarkers

Venous blood samples were obtained from patients with endometrial cancer prior to any intervention, as well as from healthy controls, at the Department of Gynecologic Oncology between January 2018 and December 2019. For both groups, all blood draws were performed in the morning between 08:00 and 10:00 h after an overnight fast of at least 8 h, in accordance with the routine preoperative laboratory protocol of our institution. All samples were transported to the Department of Biochemistry within 15 min of collection, where they were centrifuged, aliquoted, and stored at -80°C until further biochemical analysis.

The serum HE4 (BOSTER Biological Technology, Pleasanton, CA; Cat No: EK1469), Endocan (BOSTER Biological Technology, Pleasanton, CA; Cat No: EK0752), STN (Bioassay Technology Lab, Cat No: E6511Hu), and Neopterin (Bioassay Technology Lab, Cat No: E3155Hu) levels were measured in serum samples using enzyme-linked immunosorbent assay (ELISA) test kits. All ELISA kits were validated for serum measurements according to the manufacturer’s manual. Measurements were performed using an Eon. BİOTEK brand ELISA device (BioTek Instruments, Inc., Winooski, VT) in line with the instructions provided in the commercial kits. Samples were diluted based on the literature. All readings were in the linear range of the standard curve. Standards were run in duplicate; clinical samples were measured once per sample. Duplicate precision of non-zero standard points was assessed using coefficient of variation (CV%) (excluding the zero/blank standard); across assay runs, standard-duplicate CV% values were low (overall median [IQR] 6.4% [2.8–10.4]). Standard curve R^2^ values in curve-fitting reports were >0.98.

## RESULTS

The study cohort included 198 individuals, comprising 133 patients with histologically confirmed endometrial cancer and 65 healthy controls. No statistically significant differences were observed between the patient and control groups in terms of age, body weight, body mass index (BMI), or smoking status (p>0.05 for all). Among patients, most tumors were low-grade (76.7%) and endometrioid (89.5%); early-stage disease predominated (stage IA 63.2%, IB 15.0%). Clinical, pathological, and demographic characteristics of the entire cohort are presented in Table 2.

**Table 2:** Clinical, demographic, and pathological characteristics of endometrial cancer patients and healthy controls.

	Endometrial cancer Group (n=133)	Control group (n=65)	p
Age, years: median (IQR)	57 (52–68)	56 (51–66.5)	0.626
Menopausal status, n	Premenopausal: 26 Postmenopausal: 107	Premenopausal: 6 Postmenopausal: 59	0.064
Weight, kg: median (IQR)	80.0 (72.0–88.0)	79.0 (71.5–87.5)	0.386
BMI, kg/m^2^: median (IQR)	32.0 (28.9–35.6)	32.0 (31.0–37.0)	0.220
Current smoker, n (%)	33 (24.8%)	23 (35.4%)	0.121
**Grade**	**n (%)**		
Low grade (Grade 1)	102 (76.7%)	N/A	
High grade (Grade 2–3)	31 (23.3%)	N/A	
**LVSI**	**n (%)**		
Negative	101 (75.9%)	N/A	
Positive	32 (24.1%)	N/A	
**MELF pattern**	**n (%)**		
Negative	113 (85.0%)	N/A	
Positive	20 (15.0%)	N/A	
**FIGO stage^†^**	**n (%)**		
Stage IA	84 (63.2%)	N/A	
Stage IB	20 (15%)	N/A	
Stage II	8 (6%)	N/A	
Stage IIIA	4 (3%)	N/A	
Stage IIIB	2 (1.5%)	N/A	
Stage IIIC1	6 (4.5%)	N/A	
Stage IIIC2	3 (2.3%)	N/A	
Stage IVA	0 (0%)	N/A	
Stage IVB	6 (4.5%)	N/A	

BMI: body mass index; LVSI: lymphovascular space invasion; MELF: microcystic elongated fragmented pattern; FIGO: The International Federation of Gynecology and Obstetrics; N/A: not applicable to the control group; IQR: interquartile range; FIGO: International Federation of Gynecology and Obstetrics.

^†^Based on 2009 FIGO staging.

**Figure 1:**
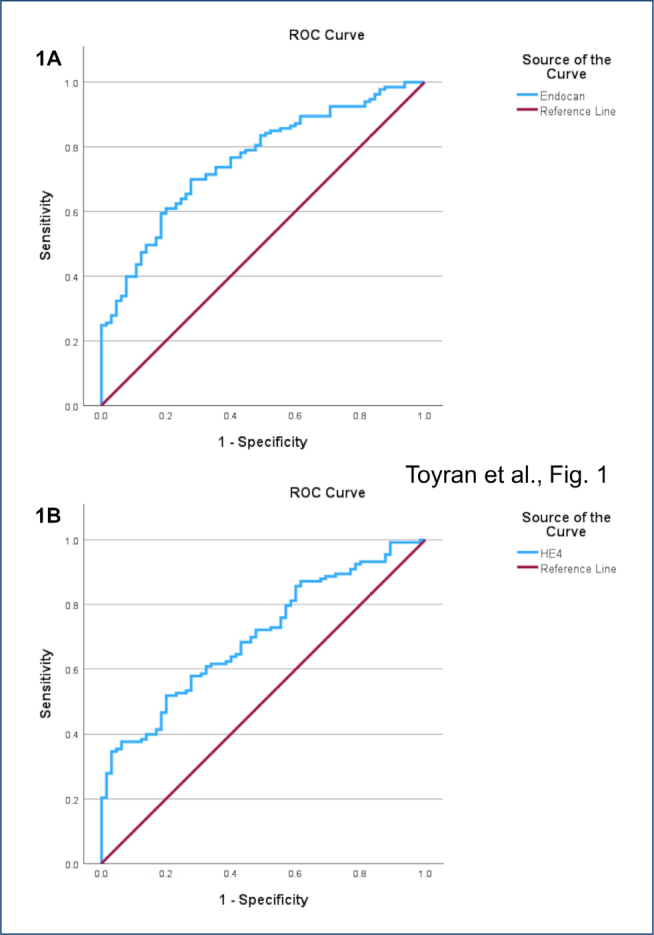
Receiver operating characteristic curves for serum endocan (A) and human epididymis protein 4 (B) distinguishing endometrial cancer versus controls.

Serum endocan levels were significantly higher in patients with endometrial cancer compared to healthy controls (153.8 [109.1–266.0] pg/mL vs. 99.9 [73.8–131.2] pg/mL; Hodges–Lehmann (HL) median difference 61.5 [40.9–86.7]; r=0.42; p<0.001). ROC analysis showed an AUC of 0.758 (95%CI 0.690–0.826; p<0.001). The optimal cut-off value was 121 pg/mL, with 69.9% sensitivity and 72.3% specificity (Figure 1A). Subgroup analysis demonstrated higher endocan levels in patients with lymphovascular space invasion (LVSI) (207.2 [123.8–313.6] vs. 147.5 [107.1–262.1] pg/mL; HL median difference 54.6 [7.4–105.7]; r=0.20; unadjusted p=0.020; Table 1). However, this association did not remain statistically significant after Benjamini-Hochberg FDR correction across the Table 1 comparisons (q=0.126). In a separare explotarory analysis, endocan levels were lower in patients with BMI ≥30 compared to those with BMI<30 (142.4 [105.2–229.6] vs. 234.2 [129.4–302.9] pg/mL; p=0.032). No significant differences were observed with respect to age, smoking status, menopausal status, tumor grade, or FIGO stage.

Similarly, serum HE4 levels were significantly higher in patients with endometrial cancer compared to healthy controls (444.6 [261.7–831.1] pg/mL vs. 285.6 [179.9–419.0] pg/mL; HL median difference 167.3 [94.2–254.7]; r=0.33; p<0.001). ROC curve analysis showed an AUC of 0.702 (95%CI 0.629–0.775; p<0.001). The optimal cut-off value was 434.6 pg/mL, yielding a sensitivity of 51.8% and a specificity of 80.0% (Figure 1B). In subgroup analyses, HE4 levels were higher in patients with LVSI compared to those without (454.1 [336.5–1,290.5] vs. 405.4 [247.7–729.8] pg/mL; HL median difference 160.9 [25.3–334.9]; r=0.20; unadjusted p=0.021; Table 1). However, this association did not remain statistically significant after Benjamini-Hochberg FDR correction across the Table 1 comparisons (q=0.126). Whereas no other significant associations were observed between HE4 levels and any clinical or pathological characteristics.

There were no statistically significant differences in serum neopterin levels between the endometrial cancer and control groups (median 3.90 vs. 3.93 nmol/L; p=0.838). Similarly, serum STN concentrations did not differ significantly between groups (median 9.30 vs. 8.64 U/mL; p=0.072). STN values were unavailable in three patients and four controls due to assay failure at the well level (invalid absorbance readings outside the quantifiable range), and repeat measurement was not feasible due to limited remaining sample volume. Given the absence of statistical significance, ROC curve analyses were not performed for neopterin and STN. Subgroup analyses showed no significant associations between neopterin or STN levels and any clinical or pathological characteristics.

## DISCUSSION

In our study, serum endocan levels were significantly elevated in endometrial cancer patients compared to healthy controls. These findings are consistent with the results of Laloglu et al., who also demonstrated significantly higher serum endocan concentrations in patients with endometrial and ovarian malignancies compared to benign and healthy controls, reporting 85% sensitivity and 97% specificity in endometrial cancer at a cut-off of 185.76 pg/mL^11^. Notably, our study expands on this by providing a larger sample size and focusing solely on endometrial carcinoma.

Similar to our findings with endocan, serum HE4 levels were significantly higher in the endometrial cancer group compared to healthy controls; however, discriminatory performance was moderate (AUC=0.702) with limited sensitivity (51.8%) and specificity (80.0%) at the selected cut-off. Meta-analyses have reported pooled sensitivities around 0.58–0.65 and pooled specificities around 0.91–0.95 for HE4 in endometrial cancer^6,7,13^. In comparison, our specificity and sensitivity were lower than pooled estimates, making our findings less compelling for diagnostic use and further supporting an exploratory interpretation.

In exploratory analyses, serum endocan and HE4 levels were higher in LVSI-positive cases; however, these associations did not remain statistically significant after correction for multiple comparisons. Although Endocan has been implicated in tumor-associated angiogenesis in prior studies^14,15,16,17^, our work measured circulating endocan only and did not assess tissue expression or vascular endothelial growth factor (VEGF)/other cytokine correlations; therefore, mechanistic conclusions cannot be drawn from these data.

In our study, no statistically significant difference was observed in serum neopterin levels between patients with endometrial cancer and healthy controls. Supporting our results, Isci Bostanci et al. also reported no significant difference in serum neopterin levels between endometrial cancer patients and healthy controls. However, they found statistically significantly higher urinary neopterin levels in the patient group compared to controls^12^. Additionally, Unuvar et al. compared preoperative serum neopterin levels in early-stage endometrial cancer patients and healthy individuals, and similarly found no statistically significant difference, further supporting the notion that serum neopterin may lack sufficient discriminatory capacity for diagnostic purposes in this setting^18^.

Similarly to neopterin, we found no statistically significant difference in the mean serum STN concentration between the endometrial cancer and control groups. To the best of our knowledge, no studies have directly compared serum STN levels between endometrial cancer patients and healthy individuals. In a prospective study of 146 patients, Hashiguchi et al. reported elevated serum STN levels in 24.7% of cases^19^. Given the limited comparative data, our findings provide preliminary evidence, though the scarce literature constrains further evaluation of its diagnostic value.

While our study provides data on circulating biomarkers in endometrial cancer, several limitations should be acknowledged. First, this was a case–control comparison against asymptomatic healthy volunteers rather than symptomatic women. Therefore, clinical applicability for triage is limited. Second, clinical samples were measured once per sample rather than in replicate; although plate-level quality control using duplicate standards was performed, sample-level intra-assay precision (CV%) could not be robustly quantified, and analytical variability may have influenced effect estimates. Third, cancer antigen 125 was not collected systematically, preventing a head-to-head comparison with an established serum marker and assessment of incremental value beyond standard clinical evaluation. Fourth, although the overall cohort exceeded the minimum sample size estimated for the primary case–control comparisons, subgroup analyses may have been underpowered. Fifth, the study was conducted at a single tertiary center, which may limit generalizability. Sixth, we measured serum biomarkers only and did not assess tissue expression or angiogenic/inflammatory mediators; thus, mechanistic inferences cannot be made. Finally, due to the cross-sectional design, prognostic endpoints such as overall survival and progression-free survival could not be evaluated.

## CONCLUSION

Our study showed significantly higher serum endocan and HE4 levels in endometrial cancer patients than in healthy controls and demonstrated moderate discrimination between groups. Given the case–control design, these findings should be interpreted as hypothesis-generating and require validation in symptomatic cohorts with benign endometrial pathology before any clinical application or risk stratification can be considered.

## Data Availability

The datasets generated and/or analyzed during the current study are available from the corresponding author upon reasonable request.
